# The p90 Ribosomal S6 Kinase (RSK) Is a Mediator of Smooth Muscle Contractility

**DOI:** 10.1371/journal.pone.0058703

**Published:** 2013-03-13

**Authors:** Mykhaylo Artamonov, Ko Momotani, Darkhan Utepbergenov, Aaron Franke, Alexander Khromov, Zygmunt S. Derewenda, Avril V. Somlyo

**Affiliations:** Department of Molecular Physiology and Biological Physics, University of Virginia, Charlottesville, Virginia, United States of America; Cinvestav-IPN, Mexico

## Abstract

In the canonical model of smooth muscle (SM) contraction, the contractile force is generated by phosphorylation of the myosin regulatory light chain (RLC_20_) by the myosin light chain kinase (MLCK). Moreover, phosphorylation of the myosin targeting subunit (MYPT1) of the RLC_20_ phosphatase (MLCP) by the RhoA-dependent ROCK kinase, inhibits the phosphatase activity and consequently inhibits dephosphorylation of RLC_20_ with concomitant increase in contractile force, at constant intracellular [Ca^2+^]. This pathway is referred to as Ca^2+^-sensitization. There is, however, emerging evidence suggesting that additional Ser/Thr kinases may contribute to the regulatory pathways in SM. Here, we report data implicating the p90 ribosomal S6 kinase (RSK) in SM contractility. During both Ca^2+^- and agonist (U46619) induced SM contraction, RSK inhibition by the highly selective compound BI-D1870 (which has no effect on MLCK or ROCK) resulted in significant suppression of contractile force. Furthermore, phosphorylation levels of RLC_20_ and MYPT1 were both significantly decreased. Experiments involving the irreversible MLCP inhibitor microcystin-LR, in the absence of Ca^2+^, revealed that the decrease in phosphorylation levels of RLC_20_ upon RSK inhibition are not due solely to the increase in the phosphatase activity, but reflect direct or indirect phosphorylation of RLC_20_ by RSK. Finally, we show that agonist (U46619) stimulation of SM leads to activation of extracellular signal-regulated kinases ERK1/2 and PDK1, consistent with a canonical activation cascade for RSK. Thus, we demonstrate a novel and important physiological function of the p90 ribosomal S6 kinase, which to date has been typically associated with the regulation of gene expression.

## Introduction

Contraction and relaxation of smooth muscle (SM), which is found in the walls of blood vessels, lymphatic vessels, bladder, uterus, the gastrointestinal, respiratory and reproductive tracts, as well as other hollow organs, play a critical role in the regulation of blood pressure, pulmonary resistance, gastrointestinal motility, urogenital and erectile function [1]. Like all muscle types, SM cells contract in response to Ca^2+^ influx through membrane channels and Ca^2+^ release from the sarcoplasmic reticulum, which drive the highly conserved cross-bridge cycle [2]. In SM, contraction is induced when Ca^2+^-bound calmodulin binds to and activates the myosin light chain kinase (MLCK), which phosphorylates the regulatory myosin light chain (RLC_20_) on Ser19, with concomitant activation of the ATPase activity of myosin [1]. Conversely, dephosphorylation of RLC_20_ by the RLC_20_-phosphatase (MLCP) inhibits contraction and induces relaxation [3], [4].

Over the past two decades, it has been shown that this relatively simple Ca^2+^/calmodulin-dependent paradigm is additionally modulated in a complex and often tissue-specific way by phenomena which are Ca^2+^-independent and which amplify the contractile response to Ca^2+^, leading to ‘Ca^2+^ sensitization’ [5], [6]. Two distinct molecular mechanisms have been reported for this pathway. The first of these invokes Ca^2+^-independent activation of kinases other than MLCK, capable of direct phosphorylation of RLC_20_. The second mechanism involves agonist-dependent down-regulation of MLCP, leading to increased contractility at constant intracellular Ca^2+^ concentration ([Ca^2+^]_i_). The release of select agonists (e.g. thromboxane A_2_), which act on G-protein coupled receptors (GPCRs), up-regulates the cytosolic GTPase RhoA, which in turn activates the Rho-associated protein kinase-ROCK. The latter phosphorylates the myosin targeting subunit (MYPT1) of the heterotrimeric MLCP, made up also of the catalytic subunit (PP1C) and a 21 kDa subunit [7], [8]. Phosphorylation of MYPT1 leads to inhibition of the phosphatase activity of PP1C, consequently sustaining RLC_20_ phosphorylation and thus enhancing the SM contractile force. Interestingly, both Ca^2+^ independent ZIPK and ILK also phosphorylate MYPT1 and suppress PP1C activity [9], [10].

Although Ca^2+^-sensitization is now recognized as a major regulatory mechanism in SM, and is targeted for such conditions as hypertension, it is also increasingly clear that additional regulatory mechanisms, both Ca^2+^-dependent and independent, must be in place in diverse SM tissues. Evidence for Ca^2+^-independent activity is seen when microcystin-LR, a phosphatase inhibitor, is added to permeabilized SM in the absence of [Ca^2+^]_i_ [11]; under these conditions the level of phosphorylation of RLC_20_ increases, resulting in contraction. It has also been shown that the blood vessels isolated from MLCK-null mice embryos at E14.5 to term and permeabilized with α-toxin, develop force in response to increase in [Ca^2+^]_i,_ [12]. Moreover, Ca^2+^-sensitization can be induced in MLCK-null vessels by addition of GTPγS and this contraction can be relaxed by the Rho-kinase inhibitor, Y-27632 [12]. Thus, the MLCK (-/-) SM seems to utilize Ca^2+^ dependent kinase(s) other than MLCK, or/and Ca^2+^-independent kinase(s) which are activated by Ca^2+^-dependent upstream signaling.

Interestingly, it has been reported some time ago that the p90 ribosomal S6 kinase (RSK2), which is typically associated with regulation of gene expression, can phosphorylate RLC_20_ *in vitro*, although the physiological consequences have never been explored [13]. The four known RSK isoforms (RSK1-4), as well as the related mitogen- and stress-activated kinases MSK1 and MSK2, display unique molecular architecture, with two distinct protein Ser/Thr kinase domains in tandem, i.e. the regulatory C-terminal kinase domain (CTKD), and the physiologically active N-terminal kinase domain (NTKD) [14], [15], [16]. The activation of RSK kinases involves several sequential phosphorylation steps, initiated by the docking of extracellular signal-regulated kinases (ERK1/2) at the C-terminus and subsequent phosphorylation and activation of CTKD, intramolecular phosphorylation of the interdomain linker at Ser380, the recruitment of active phosphoinositide-dependent kinase 1 (PDK1) to this newly phosphorylated site, and finally PDK1-dependent phosphorylation of Ser227 (RSK2 numbering) within the T-loop, with concomitant activation of the NTKD [15]. Both ERK1/2 and PDK1 kinases are known to function in SM, and have been implicated in some tissues in regulation of SM proliferation and contractility [17], [18], [19].

Since the original observation of RLC_20_ phosphorylation by RSK2 was reported, two selective inhibitors for the RSK family of kinases were discovered: the flavonoid glycoside SL0101 [20] and the synthetic compound BI-D1870 [21]. In the present study we took advantage of these reagents to investigate if RSK plays a role in SM contractility using *ex vivo* assays. We show that inhibition of RSK significantly reduces contractile response in intact SM stimulated by either high [K^+^] or the thromboxane A2 (TXA_2_) analogue U46619, which typically induces Ca^2+^-sensitization cascade through the activation of the TXA2 receptors and the RhoA/ROCK signaling cascade. Furthermore, RSK inhibition by BI-D1870 significantly reduces phosphorylation levels of both RLC_20_ and MYPT1, and suppresses agonist induced Ca^2+^-sensitized force, indicating that RSK functions in SM not only through phosphorylation of RLC_20_ but also through inhibitory phosphorylation of MYPT1. Further evidence for agonist-induced activation of RSK in SM is our finding that the TXA_2_ receptor synthetic agonist U46619 increases ERK1/2 and RSK2 Ser227 phosphorylation. Interestingly, our data also suggest that RSK up-regulation leads to the phosphorylation of the MYPT1 subunit not only at the canonical sites of Thr853 and Thr696, but also on Ser668.

## Materials and Methods

### Tissue preparation and force measurements

All procedures and protocols were approved by the Animal Care and Use Committee at the University of Virginia (Protocol #1834 for rabbit and rat, and Protocol #2796 for mice). The primary branches of the rabbit pulmonary artery were dissected, freed from connective tissue, cut into small strips (150–250 µm wide, 2–3 mm long) and mounted between two tungsten hooks on a bubble plate for force measurements [22], [23]. Following equilibration, intact strips were stimulated with high [K+] (179 mM) Hepes buffered solution or 300 nM U46619 in the presence of 25 nM, 100 nM or 1 µM BI-D1870 or matching diluent concentration, (DMSO) and the magnitude of contraction measured.. Strips were also permeabilized with 500 U/ml *Staphylococcus aureus* α-toxin [23] or 75 µM β-escin [24] in relaxing solution (G1) containing 4.5 mM MgATP and 1 mM EGTA. To deplete Ca^2+^ stores, strips were treated with 10 µM Ca ionophore A23187 (Calbiochem) for 10–15 min in G1. For Ca^2+^-activating solutions, 10 mM EGTA was used and a calculated amount of Ca^2+^-methanesulfonate was added to give the desired [Ca^2+^]. Measurements of IC_50_ for RSK and PDK inhibitors were carried out in muscle strips stimulated with pCa 6.7. Once the force reached a plateau, increasing concentrations of inhibitors or of the diluent (DMSO) were added, and the magnitude of force relaxation was recorded. The magnitude of relaxation was normalized to that of the pCa 6.7-induced force, taken as 100%.

The dependence of force developed in response to varying [Ca^2+^] was determined by activating the strips by increasing [Ca^2+^] from pCa>8. Muscle strips were preincubated with 100 nM of BI-D1870 or 30 µM GSK2334470 or the corresponding concentrations of DMSO as control, for 15 min in relaxing (G1) solution and then stimulated with Ca^2+^-activating solutions to allow for the magnitude of the force response to be measured. Force was normalized to maximal Ca^2+^-induced force (pCa 4.5) taken as 100%, or expressed as the absolute value in milli-Newtons (mN).

For Ca^2+^-sensitization experiments, the muscle strips were preincubated with the RSK inhibitor BI-D1870 (1 µM) or the PDK1 inhibitor GSK2334470 (30 µM), or corresponding concentrations of DMSO for 15 min in relaxing (G1) solution at low pCa 7.0 (agonist-sensitized force) and once the force reached a plateau, strips were Ca^2+^-sensitized by the addition of the TXA_2_ receptor agonist (U46619, 300 nM). Up to 1–2 µM of GTP was added together with the sensitizing agonist U46619 to compensate for possible loss of GTP during permeabilization. In the presence of inhibitor, the force was normalized to the magnitude of agonist-induced Ca^2+^ sensitized force on the same strip, recorded in the absence of inhibitor and taken as 100%. Tissue samples were frozen in relaxing solution, at the peak of agonist activation or maximal Ca^2+^ stimulation, in the presence and absence of inhibitors for biochemical assays for RLC_20_ phosphorylation on Ser19 and MYPT1 on Thr853, as well as RSK2 phosphorylation on Ser227, MYPT1 on Ser668, ERK1/2 on Ser217/221, and PDK on Ser241. In some experiments, unphosphorylated, mono- and di-phosphorylated RLC_20_ were separated on urea gels.

For experiments designed to evaluate the contribution of Ca^2+^-independent kinases to contraction of SM in the absence of MLCP activity, the following protocol was used: following permeabilization with β-escin (75 µM) to allow for the penetration of the microcystin-LR, the strips were preincubated with of BI-D1870 (1 µM), or the corresponding concentration of DMSO for 5 min in relaxing (G1) solution, plus 1 µM calmodulin for 15 min followed by addition of microcystin-LR (10 µM). Temperature was 22–24°C. After the force reached the plateau, the strips were snap-frozen in 10% TCA in acetone at −80°C for further biochemical studies. The force was normalized to agonist-induced Ca^2+^ sensitized force, taken as 100%.

For evaluation of the effect of BI-D1870 and GSK2334470 on crossbridge cycling, 0.2 mm diameter strips of mouse ileum were permeabilized with 0.5% Triton X-100 in G1 solution in the presence of myokinase inhibitor Ap5A, which inhibits ATP synthesis, (50 µM) for 20 mins. Following washing off Triton and treatment with A23187, an ionophore that depletes Ca^2+^-stores (10 µM) for 15 mins, the muscles were washed with ATP-free “rigor” solution with Ca^2+^ and irreversibly phosphorylated by endogenous MLCK with 1 mM ATPγS as published previously [25]. Upon extensive washing in Ca^2+^- and ATP-free solution, the muscle strips were activated with 4.5 mM of MgATP in the absence of Ca (G10) with our without BI-D1870 or GSK2334470. The half-time of the rate of force development was used as an index of the actomyosin ATPase rate.

### Tissue screen and western blots

SM tissues and brain were harvested from rabbits and mice, and screened for expression of RSK1, RSK2 and RSK3. Tissues and cultured cells were lysed in lysis buffer (1% Triton X-100, 150 mM NaCl, 1 mM EDTA, 50 mM Tris-HCl pH 7.5, 1 mM dithiothreitol, 1% protease inhibitor cocktail (SIGMA) for mouse tissues and 1% SDS, 300 mM NaCl, 50 mM Tris-HCl pH 7.5 for all other samples), subjected to SDS-PAGE, transferred to polyvinylidene difluoride membrane (Millipore) and visualized using the Odyssey System (Li-Cor, Lincoln, NE). To examine the phosphorylation of MYPT1 and RLC_20_, rabbit pulmonary artery SM strips were treated as described above. Following the stimulation protocols, muscle strips were immediately frozen by immersion in −80°C 10% (wt/vol) trichloroacetic acid in acetone and stored at −80°C. Frozen strips were then washed in acetone and dried, homogenized in sample buffer in a glass-glass, hand-operated homogenizer and phosphorylation was estimated by SDS-PAGE and Western blotting. For imaging, the membranes were blocked with the Odyssey blocking buffer. The membranes were subjected to anti- RSK1 (1∶500), RSK2 (1∶500), total MYPT1 (1∶2,000), phospho-MYPT1 (Thr668) (1∶1,000), phospho-MYPT1 (Thr853) (1∶1,000), phospho-MYPT1 (Thr696) (1∶1,000) and phospho-RLC_20_ (1∶500), phospho-RSK2 (Ser227) (1∶500), RSK3 (1∶500), total RLC_20_ (1∶5000) antibodies also: ERK1/2, phospho-PDK1 (Ser241) (1∶500). Antibodies were diluted in Odyssey blocking buffer. The membranes were washed in TBS with 0.05% Tween 20. Primary antibodies were visualized using secondary antibodies conjugated to either Alexa 680 or IRDye800 for Odyssey imaging.

### Preparation of GFP-ZIP kinase γ phosphate-linked sepharose

Gamma phosphate linked ATP sepharose resin was prepared as previously published [26]. The resin was mixed with HEK293 cell extract expressing full length ZIPK fused to GFP. After washing, the resin was divided and eluted with the BI-D1870 (50 µM) or 100 mM ATP. The 100 mM ATP represents a maximal signal while the minimal signal is the blank buffer with the BI-D1870 diluent DMSO.

### Preparation of recombinant RSK2

DNA fragment encoding amino acids 44–728 of murine RSK2 was amplified by Phusion polymerase and cloned into pFastBacHTb vector using BamHI and SalI sites. Recombinant virus was generated with Bac-to-Bac system and amplified in Sf-9 cells. For RSK2 overexpression, two 500 ml cultures of Sf-9 cells were infected with 5 ml of viral stock. Cells were harvested after 60 hrs and frozen at −80°C. Cell pellets were thawed in 100 ml of lysis buffer (50 mM Tris pH 8.0, 500 mM NaCl, 5 mM 2-mercaptoethanol and 1% Igepal CA-630) and homogenized in Dounce homogenizer. Cell lysate was clarified by centrifugation for 35 min at 100,000 g and subsequently incubated for 1 hr with 2 ml His-Select resin. After extensive washes the protein was eluted and further purified with size-exclusion chromatography over Sephadex 200 column. His-tag was not removed.

### Reagents

GSK 2334470 and SL0101 were purchased from Tocris Bioscience (Bristol, UK); BI-D1870 from Enzo Life Sciences (USA); Microcystin-LR and U46619 from Calbiochem (La Jolla, CA, USA); β-escin from Sigma (USA). The RSK1, RSK2, MYPT1, phospho-MYPT1 (Thr668), phospho-MYPT1 (Thr853 and Thr696), and phospho-RLC_20_ antibodies were purchased from Cell Signaling Technology, MA, USA; phospho-RSK2(Ser227) and RSK3 from Santa Cruz Biotechnology; RLC_20_ from Sigma; ERK1/2 and phospho-PDK1(Ser241) antibodies from Thermo Scientific, Il, USA, secondary antibodies from Invitrogen or Li-Cor, Lincoln, NE, for Odyssey imaging.

### Data analysis

Force responses to treatments were fit using GraphPad Prizm 5.0 software (GraphPad Software, Inc., CA). The significance of the difference between curves was determined using two-way analysis of variance. The P values for differences between curves are presented on each graph. Mean values and standard error of the mean (S.E.M.) were obtained from 3–8 independent experiments for each condition. Statistical significance of group differences was assessed using Student's t-test. The level of significance was set at p<0.05.

## Results

### Identification of RSK isoforms in rabbit and mouse smooth muscle tissues

Given the reported observation that the RSK2 isoform can phosphorylate RLC_20_ *in vitro* [13], we wondered if RSK kinases play a physiological role is SM contractility. We began by determining which of the four RSK isoforms might be functional in SM. Of the four known isoforms, RSK4 is the least abundant, and in the few tissues where it is present, it is constitutively active [27]. Therefore, we ruled out this isoform. To search for the remaining proteins we used three specific commercial antibodies for RSK1, RSK2 and RSK3. As a control we used recombinant RSK2 protein, and as expected only the anti-RSK2 antibody reacted with this sample (Figure S1A). Tissue screens for the three RSK isoforms were carried out in rabbit and mouse vascular beds and the gastrointestinal tract. The RSK3 isoform was not detected in any rabbit tissues (data not shown), which may reflect poor reactivity of the RSK3 antibody. Only very weak signals for RSK3 were detected in mouse bladder, portal vein and thoracic aorta (data not shown). In contrast, RSK2 was detected in all SM tissues tested while RSK1 was detected in rabbit but not mouse SM, except for mouse brain (mouse data not shown). The antigenic amino acid sequence of RSK1 is identical for rabbit, mouse and human (Figure S1C), and therefore the lack of a positive result in mouse is not due to differences in the antigenic sites for the two species. Although our data strongly suggest that the isoform active in SM is RSK2, the definitive clarification of which RSK isoform(s) play a role in SM contractility remains to be determined. Therefore, throughout the paper we use the generic term RSK when referring to the target kinase.

### Inhibition of RSK suppresses high [K^+^]- and U46619-induced force in intact SM

**Figure 1:**
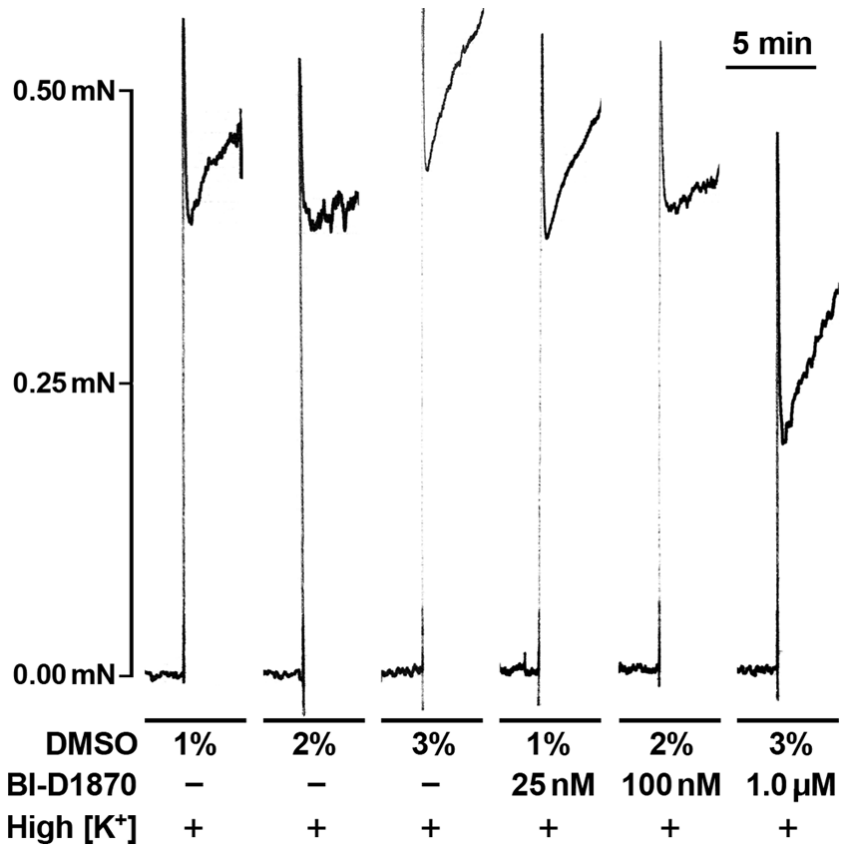
The RSK inhibitor BI-D1870 inhibits the contractile response to high [K^+^] stimulation of intact rabbit pulmonary artery. High [K^+^] (179 mM) stimulation was carried out in the presence of 25 nM, 100 nM and 1 µM BI-D1870 and on paired muscle strips the corresponding concentrations of the diluent DMSO were used as indicted. 1 µM BI-D1870 significantly inhibited both the phasic and tonic component of contraction.

**Figure 2:**
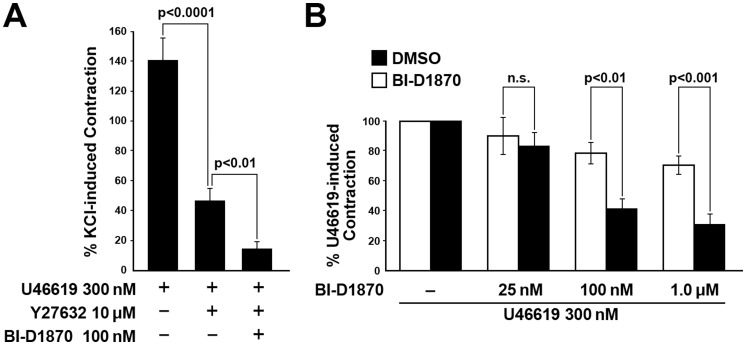
Inhibition of ROCK does not inhibit the ability of the RSK inhibitor BI-D1870 to suppress the high [K^+^] contraction and BI-D1870 significantly suppresses U46619-induced force in intact pulmonary artery. (**A**) Pretreatment of intact SM with the ROCK inhibitor, Y-27632 or Y-27632 plus BI-D1870 prior to stimulation with U46619. Force responses were normalized to the KCL-induced contraction taken as 100%. (**B**) Pretreatment of intact SM with 25 nM, 100 nM and 1 µM BI-D1870 prior to stimulation of U46619.

We first examined whether the BI-D1870 compound, which is a selective inhibitor for the RSK family of Ser/Thr kinases [28], [29] has an effect on SM contractility. Intact strips of rabbit pulmonary artery were stimulated with high [K^+^] in the presence of 25 nM, 100 nM and 1 µM BI-D1870, or the DMSO diluent. The initial large phasic component of force was significantly suppressed at the 1 µM BI-D1870 concentration (Figure 1). We then analyzed changes in U46619-induced contraction in the presence of the Rho kinase inhibitor Y-27632 and/or BI-D1870 in intact SM. High [K^+^]-induced contraction was used as a reference (i.e. 100%) to assure that the magnitude of U46619-induced contraction was significant and to compare magnitudes of contraction among different treatments. As a result, addition of Y-27632 together with BI-D1870 prior to activation with U46619 (300 nM) inhibited the contraction to a greater extent that Y-27632 alone (Figure 2A), suggesting that the inhibitory effects of the BI-D1870 are not due to the inhibition of ROCK. Contraction in response to stimulation of intact muscle with 300 nM of U46619 was suppressed by 100 nM BI-D1870, (Figure 2B). These findings strongly suggest a physiological role for RSK kinases in SM contractility, but because of the complexity of the signaling in intact SM involving ion channels, second messengers and Ca^2+^, we focused specifically on Ca^2+^-dependent and independent mechanisms, as well as RhoA-mediated Ca^2+^ sensitized force, using permeabilized SMs.

### RSK mediates Ca^2+^-induced SM contractile force by increasing phosphorylation of both RLC_20_ and MYPT1

**Figure 3:**
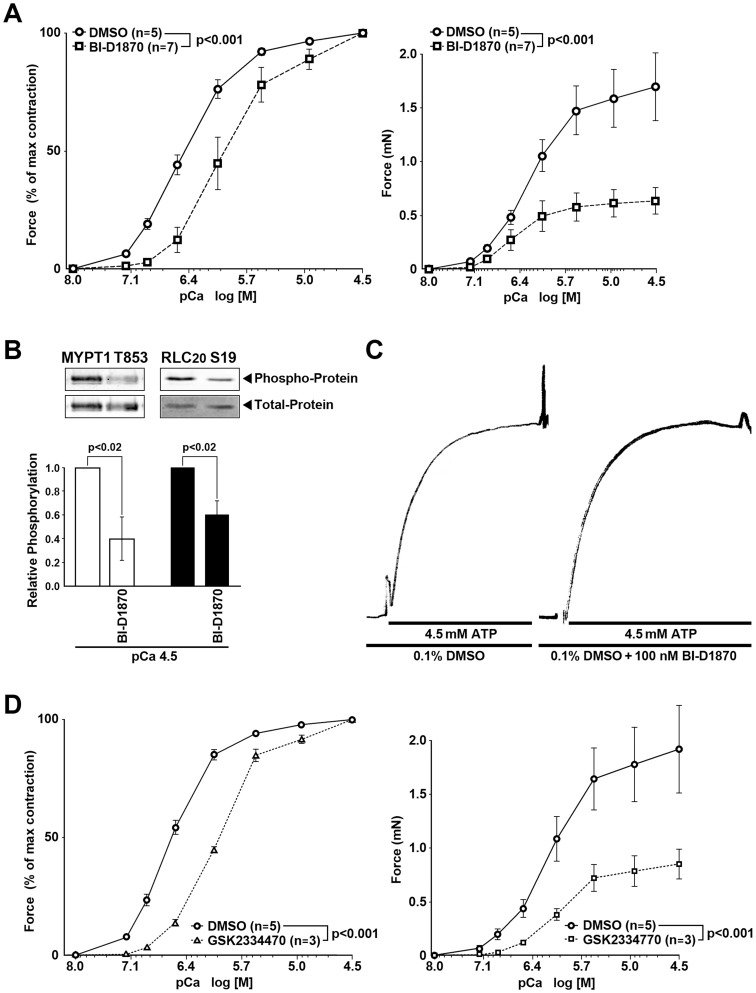
RSK and PDK1 contribute to Ca^2+^-induced force, MYPT1 and RLC_20_ phosphorylation in α-toxin permeabilized rabbit pulmonary artery SM. RSK inhibitor, BI-D1870 is without effect on the actomyosin ATPase. (A) RSK inhibitor, BI-D1870 (100nM), right shifted the pCa^2+^-force relationship and decreased the maximal force (milli-Newtons, mN) at each intracellular Ca^2+^ concentration (pCa) compared to the Control diluent, DMSO. (B) BI-D1870 (100 nM) decreased phosphorylation of MYPT1 Thr853 (n = 3) and RLC_20_ phosphorylation at Ser19 (n = 4) at maximal Ca^2+^-induced (pCa 4.5) force. (C) BI-D1870 (100 nM) was without effect on the ATP-induced rate of force development in Triton permeabilized mouse ileum SM with pre-thiophosphorylated light chains and depleted of ATP. T1/2 = 4.8±1.2 sec vs 5.0±1.3 sec in the Control (DMSO) vs BI-D1870 respectively, p = ns, n = 3. Thus, the inhibitor effects of BI-D18670 are not due to inhibition of the actomyosin ATPase. (D) The PDK1 kinase inhibitor GSK 2334470 (30 µM) also suppressed the pCa^2+^-force relationship and reduced the absolute force (mN).

Using strips dissected from rabbit pulmonary arteries and permeabilized with α-toxin (see Materials and Methods), we directly measured the contractile force generated by an increase in [Ca^2+^] in the absence of inhibitors and then in the presence BI-D1870 (100 nM). In the presence of the inhibitor, we observed two distinct effects: the onset of contraction was shifted to higher [Ca^2+^] and the maximum force was significantly reduced (∼65%) as compared for the control samples treated only with the diluent DMSO (Figure 3A). We also determined the IC_50_ value for BI-D1870, which was ∼17 nM (Figure S2). The RSK inhibitor SL0101 [20] (30 µM) also reduced Ca^2+^-induced force, but due to its poor solubility, it has not been possible to credibly quantify the data (Figure 2).

Although we expected that the observed effects of BI-D1870 may be due to suppression of direct phosphorylation of the RLC_20_, the contractile force can be also affected negatively by up-regulation of the MLCP activity. Therefore, we checked if the effect of the RSK inhibitor is correlated with a decrease in phosphorylation of RLC_20_ or/and MYPT1. Using phospho-specific antibodies, we determined that in permeabilized rabbit arteries preincubated for 15 min at pCa 4.5 in the presence of 100 nM BI-D1870, phosphorylation levels of RLC_20_ on Ser19 and of MYPT1 on Thr853 (the main ROCK inhibitory site) were both significantly decreased (Figure 3B). The BI-D1870-induced decrease in Ca^2+^-mediated RLC_20_ phosphorylation may reflect its direct or indirect phosphorylation by RSK or is secondary to the decrease in MYPT1 Thr853 phosphorylation and increased phosphatase activity.

To rule out the possibility that the inhibitory effects of BI-D1870 or GSK2334470 on contraction are due to inhibition of actomyosin ATPase activity, we measured the rate of ATP-induced force in Triton permeabilized SM with thiophosphorylated RLC_20_ in Ca^2+^ free rigor solution (ATP free) The presence of BI-D1870 (100 nM) (Figure 3C) or GSK2334470 (30 µM) (data not shown) had no significant effect on the cycling of phosphorylated crossbridges (t1/2 = 4.8±1.2 sec Control (DMSO) vs 5.0±1.3 sec BI-D1870, p = ns, n = 3 and t1/2 = 5.1±0.8 Control (DMSO) vs 5.4±0.2 GSK2334470, p = ns, n = 3).

The constitutively active PDK1 kinase is required for upstream activation of RSK [15], [30]. To clarify if PDK1 is also involved in the RSK-mediated SM contractility, we treated the α-toxin permeabilized pulmonary artery SM with the selective PDK1 inhibitor, GSK2334470 (30 µM). The treatment resulted in a significant decrease in the contractile force in response to [Ca^2+^] (Figure 3D) along with reduction in phosphorylation of MYPT1 on Thr696 to ∼50%, on Thr853 to ∼75%, and concomitant reduction in phosphorylation of RLC_20_ at Ser19 to ∼60% as compared to control experiments (data not shown). These results conclusively implicate the involvement of canonical regulatory mechanisms for RSK in SM contractile regulation.

### The MLCP-independent activity of RSK in smooth muscle

**Figure 4:**
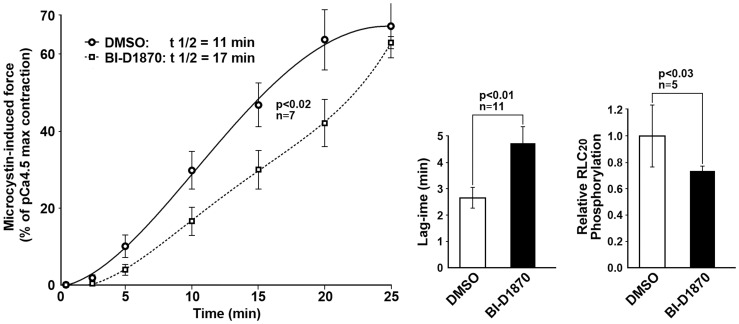
RSK functions as a Ca^2+^ independent kinase to increase force and RLC_20_ phosphorylation. RSK inhibition increases the time to the onset of force and the rate of force development (t1/2) induced by the MLCP (PP1C) inhibitor microcystin-LR (10 µM) in the absence of Ca^2+^(pCa 9.0) The relative phosphorylation of RLC_20_, measured at 25 min following the addition of microcystin, was decreased in the presence of BI-D1870 (1 µM).

The potent, irreversible inhibitor of MLCP, microcystin-LR (microcystin), is routinely used as a reagent that allows the study of signaling phenomena independent of MLCP. In a control experiment using a rabbit pulmonary artery permeabilized with β-escin, microcystin induces a slow contraction at pCa 9.0 (1 nM Ca^2+^), i.e. with [Ca^2+^] well below normal contraction threshold (pCa 7.0), which attests to the phosphorylation of RLC_20_ catalyzed by Ca^2+^-independent kinases. We asked what effect BI-D1870 had under the conditions of this experiment. The addition of the inhibitor significantly increased the lag time to the onset of force measured at the intercept of the baseline with a straight line fitted to the rising phase (2.7 min±0.4 vs 4.7 min±0.7, p<0.01 n = 11); and the rate of force development (t _½_ = 11 vs 17 min, p<0.02) (Figure 4). BI-D1870 treatment also decreased microcystin-induced RLC_20_ phosphorylation (Figure 4), measured at 25 min following addition of microcystin and normalized to the value in the diluent (1.0±0.24 vs 0.7±0.06, p<0.03, n = 5). This may be an underestimate of Ser19 phosphorylation as microcystin treatment leads to phosphorylation of Ser19 and Thr18 and the phospho-Ser19 specific antibody may not recognize the RLC_20_ phosphorylated at both Ser19 and Thr18 [31]. Subsequent addition of Ca^2+^ to the sample containing both microcystin and BI-D1870 resulted in a further increase of force to 77±5.5% vs 79±4.3% for the diluent expressed as% maximal contraction with pCa 4.5 measured prior to treatment with microcystin providing additional evidence that BI-D1870 did not inhibit MLCK.

**Figure 5:**
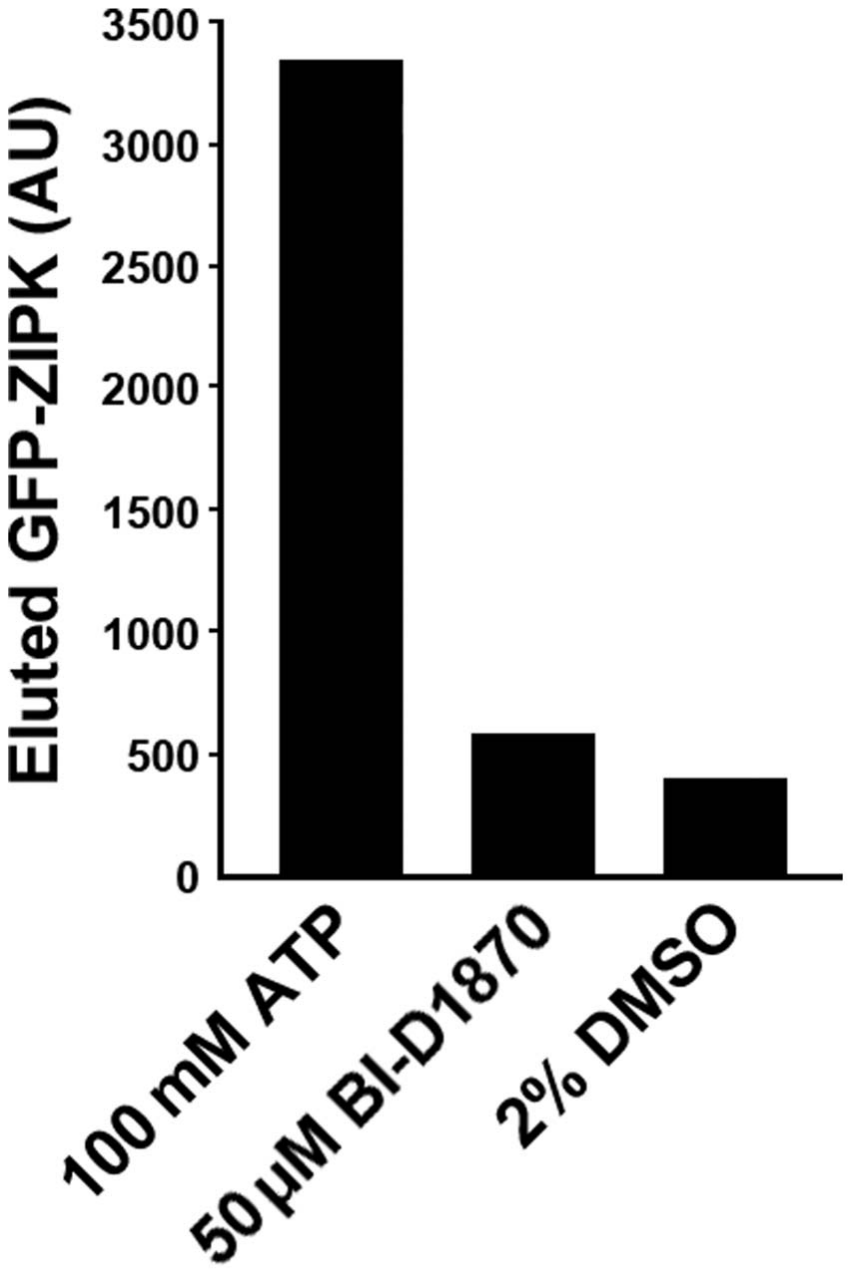
The RSK inhibitor BI-D1870 does not function through inhibition of ZIP kinase. The elution of full length GFP-ZIPK off of γ phosphate linked ATP resin in the presence of 100 mM ATP, 50µM BI-D1870 or blank buffer (2% DMSO) is shown. We used 100 mM ATP because of the dense resin concentration (10 mM). 100 mM ATP gives a maximal signal. The small signal given by BI-D1870 above background (2% DMSO) is not considered relevant.

To address the possibility that the inhibition of the microcystin-induced contraction by BI-D1870 was due to the inhibition of the Ca^2+^ independent ZIP kinase [32], we tested the ability of BI-D1870 (50 µM) to elute full length GFP-ZIPK bound through its nucleotide binding site to a γ-phosphate linked ATP-sepharose column [26]. While 100 mM ATP (used for the maximal signal) readily eluted GFP-ZIPK from the column, BI-D1870, a known potent ATP-competitive inhibitor of RSK isoforms [28] was without effect (Figure 5). Thus it is highly unlikely that the BI-D1870 inhibition of the Ca^2+^ independent microcystin-induced contraction is through inhibition of ZIP Kinase.

### RSK mediates agonist-induced, Ca^2+^-sensitized force

We next asked if agonist stimulation might also up-regulate RSK and mediate SM contractility in a Ca^2+^-independent manner, targeting MYPT1 and consequently MLCP. We used an assay involving the synthetic agonist U46619 for the thromboxane A2 (TXA_2_) receptor.

**Figure 6:**
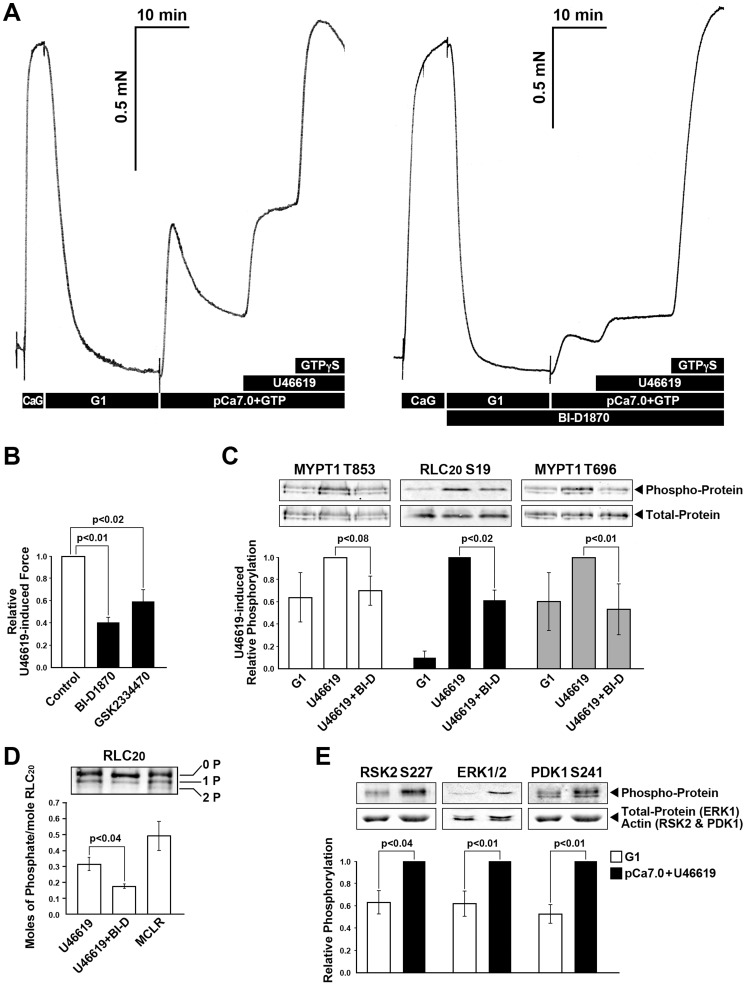
RSK and MEK/ERK signaling pathways contribute to TXA_2_-induced Ca^2+^ sensitized force and MYPT1 and RLC_20_ phosphorylation in α-toxin permeabilized rabbit pulmonary artery SM. (**A**) U46619 (300 nM) induced Ca^2+^ sensitized force is suppressed in the presence of RSK inhibitor, BI-D1870 (100 nM). Subsequent addition of GTPγS, as a measure of the remaining component of Ca^2+^ sensitized force, reached the same magnitude of force but was proportionately greater in the presence of the RSK inhibitor BI-D1870 than in its absence. (**B**) Summary of panel A along with an effect of PDK1 kinase inhibitor GSK2334470 (30 µM) on U46619-induced force. (**C**) RSK inhibitor, BI-D1870 (1 µM) significantly inhibited the U46619 (300 nM) increased phosphorylation of MYPT1 Thr853 (n = 3), RLC_20_ phosphorylation at Ser19 (n = 5) and MYPT1 Thr696 phosphorylation (n = 5). (**D**) Unphosphorylated (0P), singly (1P) and doubly phosphorylated (2P) RLC_20_ from SM samples stimulated with U46619 with and without BI-D1870 as in panel C were separated on urea gels. A positive control for doubly phosphorylated RLC_20_ was a β-escin treated SM stimulated with microcystin. (**E**) U46619 stimulation increased the phosphorylation of proteins that regulate RSK activation; RSK2 Ser227 (n = 8), ERK1/2 (n = 7) and PDK1 Ser241(n = 15).

The U46619-induced force in the permeabilized rabbit pulmonary artery SM at constant [Ca^2+^] (pCa 7.0) was suppressed in the presence of 100 nM BI-D1870 by more than ∼50% compared with untreated control (Figure 6A and B). When GTPγS was added to the control sample and the sample with BI-D1870, the same level of contractile force was recorded, attesting to the fact that maximal activation of RhoA by GTPγS compensated for the BI-D1870 mediated inhibition of Ca^2+^-sensitized force (Figure 6A). Phosphorylation of RLC_20_ at Ser19 as well as MYPT1 at both the Thr853 and Thr696 inhibitory sites, associated with U46619 induced force, was significantly decreased in the presence of BI-D1870 (Figure 6C). Because the phospho-specific RLC_20_ antibody is less sensitive when both the Ser19 and Thr18 sites are phosphorylated [31], urea gels were used to separate singly and doubly phosphorylated light chains (Figure 6D). U46619-induced Ca^2+^ sensitized force resulted in only singly phosphorylated RLC_20_ and this decreased with BI-D1870 treatment confirming that RSK activation results in phosphorylation at the Ser19 site (Figure 6C). Stimulation of the permeabilized rabbit pulmonary artery SM with U46619 at constant [Ca^2+^] (pCa 7.0) also induced an increase in phosphorylation of RSK2 at Ser227 and its upstream regulator ERK1/2 (Figure 6E) indicating activation of RSK occurring through the canonical MEK/ERK pathway.

Given that the MEK/ERK pathway also depends on the constitutive activity of the PDK1 kinase, we asked if inhibition of PDK1 might alter contraction of SM. An increase in phosphorylation of PDK1 was also detected after stimulation with U46619 (Figure 6E). When PDK1 was inhibited by its selective inhibitor GSK2334470 (30µM), U46619 induced Ca^2+^-sensitized force was suppressed by ∼40% compared with untreated rabbit pulmonary artery SM (Figure 6B) along with concomitant decrease in MYPT1 phosphorylation at both Thr853 to (40±3% as compared to control, n = 3, p<0.01) and at Thr696 to (55±25% as compared to control, n = 3, p<0.01) (data not shown). Taken together, these series of results suggest involvement of RSK under the MEK/ERK pathway in the Ca^2+^-sensitization pathway of SM regulation.

### Potential phosphorylation of MYPT1 on ser668 by RSK

**Figure 7:**
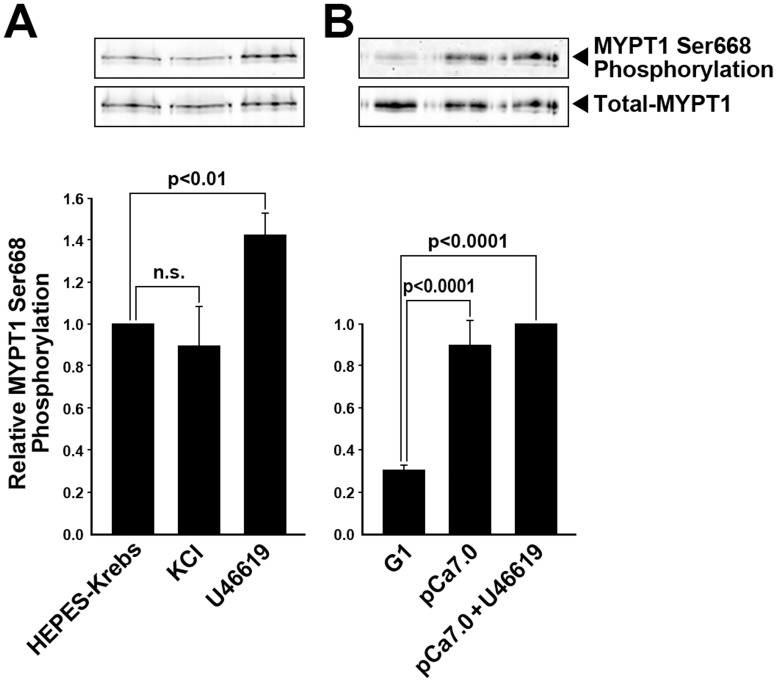
MYPT1 phosphorylation at Ser668 is increased upon stimulation with the TXA_2_ analogue, U46619, in permeabilized and intact pulmonary artery SM but not by high [K^+^] stimulation in intact SM. (**A**) U46619 (300 nM) (n = 3) but not high [K^+^] stimulation increases phosphorylation of MYPT1 Ser668 in intact pulmonary artery smooth muscle. (**B**) Increasing Ca^2+^ to pCa7.0 with and without U46619 (300 nM) induced Ca^2+^ sensitized force in α-toxin permeabilized SM significantly increased phosphorylation of MYPT1 at Ser668 compared to control muscles in G1 solution pCa<8.0 (59±9.6%, n = 10, p<0.01).

An analysis of the amino acid sequence of MYPT1 revealed a third potential RSK phosphorylation motif (RRXS/T) which includes Ser668. We found that in intact pulmonary arteries, the phosphorylation level of MYPT1 at Ser668 increased by 42±10.5%, n = 3, p<0.01; following stimulation with U46619, but no change was detected with high [K^+^] stimulation (Figure 7A). The phosphorylation level of Ser668 was also increased by ∼45% in α-toxin permeabilized SM strips stimulated by Ca^2+^ (pCa 7.0) and U46619 (Figure 7B). The functional significance of this phosphorylation remains to be determined.

## Discussion

Dysregulation of SM contractility is an underlying factor in several pathophysiological phenomena, such as hypertension, asthma, bowel disease, cerebral vasospasm and others [33]. Given the prevalence of hypertension alone, and the fact that many patients do not respond to available medications, there is an ongoing search for alternative therapies targeting specific signaling pathways. Detailed characterization of all physiological regulatory pathways in SM tissues is a prerequisite for identification of suitable targets for drug discovery. To date, only the ROCK kinase, involved in Ca^2+^-sensitization has been considered a valid drug target, but the ubiquitous distribution of ROCK and its many physiological functions raise concerns about side effects. Nevertheless, fasudil a potent ROCK inhibitor (currently in clinical trials in the US), is used in Japan to treat cerebral vasospasm [34]. A possible involvement of other kinases in the Ca^2+^-induced contraction and sensitized force opens alternative avenues for drug discoveries.

It has long been recognized that kinases other than MLCK may operate in SM, such as the Ca^2+^-independent kinases zipper-interacting protein kinase (ZIPK) [32], [35] and the integrin-linked kinase (ILK) [36], [37]. An interesting variation of this scheme involves Ca^2+^-independent kinases, such as proline-directed kinases [38], [39], [40], acting indirectly on RLC_20_, by phosphorylating and up-regulating MLCK.

We were intrigued by a report that the p90 ribosomal S6 protein kinase (RSK2) was also able to phosphorylate RLC_20_ *in vitro*. The involvement of RSK kinases in SM contraction has never been investigated before, although their presence in SM is relatively well documented [41]. Similarly, the presence in SM of ERK1/2 kinases, and the constitutively active PDK1 kinase has also been established [19], [33], [42], [43]. However, to our knowledge no direct study has implicated RSK in physiological SM contractility. To inhibit the RSK activity we used two recently described selective inhibitors, the naturally occurring, rare flavonol glycoside kaempferol-3-O-(3”,4”-di-O-acetyl-α-L-rhamnopyranoside) (SL0101) [20] and the unrelated synthetic compound BI-D1870 derived from the pyrido[2,3-*d*]pyridimidine group of Src inhibitors [21]. A recent study of a large panel of protein kinases showed that neither SL0101 nor BI-D1870 has any inhibitory effect on the two principal kinases in SM contractility pathways, i.e. MLCK or ROCK, [29]. Thus, the effects described in our study are highly unlikely to be due to off-target phenomena. The use of selective kinase inhibitors circumvented the need for a time-intensive gene silencing procedure, and we were able to use fresh pulmonary artery strips for technically easy, direct force measurements. We next plan to generate RSK2/RSK3 null SM cells to further explore the contribution of RSK to vascular contractility and basal tone.

Our data show, for the first time, that RSK kinases are indeed involved in SM contractility. We found, that in the α-toxin permeabilized rabbit pulmonary artery the RSK inhibitor BI-D1780 significantly decreased the contractile force response to [Ca^2+^], and shifted the onset of contraction to pCa 7.1 from pCa of 8.0. Furthermore, BI-D1870 also inhibited contractions elicited by high [K^+^] and U46619 in intact SM demonstrating that the observed effects in permeabilized SM are not merely due to loss of cytoplasmic components. Our findings were not expected, because RSK kinases have been associated primarily with signaling downstream of growth factors and receptor tyrosine kinases and are thought to play key roles in cell cycle progression, cell growth, survival and proliferation [30]. To dissect the specific pathway through which RSK potentiates Ca^2+^-induced contraction, we analyzed the phosphorylation levels of both RLC_20_ and MYPT1 in the presence of inhibitor. Both proteins were phosphorylated on the canonical regulatory sites, Ser19 and Thr853 respectively, at a significantly higher level in the absence of inhibitors, strongly suggesting that RSK simultaneously up-regulates myosin and down-regulates MLCP, resulting in a significant contribution to contractility. We also demonstrated that the mechanism of action of the RSK inhibitor is not through inhibition of the SM actomyosin ATPase.

It is not clear at this point in what way increased Ca^2+^ concentration may activate RSK. Again, this observation is not *per se* novel, as it has been already reported that in growth arrested cultured vascular SM cells angiotensin II increased RSK activity which was inhibited (directly or indirectly) by chelation of intracellular Ca^2+^ [41]. Several options linking Ca^2+^ to RSK activation have been discussed in literature [44], [45]. One distinct possibility is that another Ca^2+^-dependent kinase functions up-stream of RSK. For example, calmodulin-dependent kinase I (CaMKI) has been shown under some conditions to mediate the activation of the ERK1/2 kinases, which prime RSK for phosphorylation and activation by PDK1, by elevated intracellular [Ca^2+^] [46]. The precise pathway by which RSK may be linked to [Ca^2+^]-induced signaling in vascular SM is under investigation in our group.

A new, exciting discovery reported in this paper is that BI-D1870 has a significant impact on the phosphorylation of MYPT1, suggesting that inhibition of MLCP may constitute an important mechanism by which RSK potentiates the contractile force. Moreover, we determined that in response to stimulation by the thromboxane A2 analogue U46619 at constant pCa, RSK phosphorylates MYPT1 at Thr696 and Thr853, the two known inhibitory sites. Both these sites are targets for the RhoA-dependent ROCK kinase and their phosphorylation accounts for the Ca^2+^-sensitization effect [47], but both also conform to the established RSK-consensus phosphorylation sequence motif RRX(S/T). This Ca^2+^-independent mechanism is also consistent with the decreased sensitivity to Ca^2+^ in inhibitor treated samples (Figure 3), as higher Ca^2+^ concentrations are required to trigger contraction in the presence of up-regulated (or rather uninhibited) activity of MLCP. Thus, RSK may be recruited in agonist-stimulated Ca^2+^-sensitization, through a pathway canonically identified with ROCK, as well as acting as a Ca^2+^-independent MLCK.

One of the interesting outcomes of our study is the identification of the third potential phosphorylation site in MYPT1, i.e. Ser668. This is one of several sites in MYPT1 reported by Cell Signaling Technology using LC-MS/MS platform to be phosphorylated in a number of human cancers [48]. Insulin has also been shown to result in MYPT1 phosphorylation at Ser668 [49], although neither the function of this phosphorylation nor the responsible kinase have been identified. Protein kinase G (PKG1α) known to selectively phosphorylate MYPT1 isoforms that include a C-terminal leucine zipper has also been shown to phosphorylate MYPT1 on Ser668 *in vitro* [50]. This is not surprising, as PKG, PKA and RSK kinases all belong to the AGC kinase group and have similar phosphorylation motifs. However, cyclic nucleotides lead to Ca^2+^ desensitization and relaxation of SM [6], whereas activation of RSK is expected to result from agonist activation that increase ERK activation leading to contraction of SM [17].

Perhaps the functional outcome of Ser688 phosphorylation may depend on whether other sites on MYPT1 are phosphorylated, and if the combined structural effects lead to activation or inhibition of MLCP activity on myosin. Details of this mechanism remain to be determined.

The activation of RSK kinases involves several sequential phosphorylation steps, initiated by the docking of extracellular signal-regulated kinases (ERK1/2) at the C-terminus with subsequent phosphorylation and activation of CTKD. Our findings that U-46619-induced Ca^2+^ sensitized force, MLC20 and MYPT1 phosphorylation correlates with an increase in phosphorylated ERK1/2, RSK2 S227 and PDK1 S241 are all consistent with agonist-induced RSK activation. However, the original *in vitro* observations that p42MAPK suppressed the ability of RSK2 (in the absence of CTKD) to phosphorylate RLC_20_ but not a C-terminal peptide of human 40S ribosomal protein S6 [11] is inconsistent with our findings and hypothesis. This difference may be due to the use of *in vitro* vs *in vivo* protocols and if so, reflects the importance of intracellular compartmentalization and concentrations of signaling molecules. One possible further explanation of the discrepancy in the findings is that *in vivo* activated RSK may preferentially phosphorylate MYPT1 rather than directly phosphorylate RLC_20_.

While the activation of the ERK1/2 pathway by the thromboxane A2 receptor in SM has been reported before [51], the physiological consequences for SM contractility are described here to our knowledge for the first time. Inhibition of ERK1/2 and p38MAPK have been previously shown to suppress the Ca^2+^-independent protein kinase activity observed with microcystin-induced force in rat ileal SM at 1 µM microcystin [52]. At 10 µM microcystin, where PP1C was maximally inhibited, ERK1/2 and p38MAPK inhibitors had no effect. In this same study, inhibition of ROCK with Y-27632 or ZIPK with SM1 peptide had no effect, prompting the authors to suggest that ERK1/2 and p38MAPK are contributing to ILK-mediated inhibition of MLCP activity. Based on the present findings, we suggest that RSKs are the physiological ERK1/2 substrates, perhaps in addition to ILK. An interesting difference between the experiments utilizing RSK inhibitors (our study) and those using ERK1/2, p38MAPK and PKC inhibitors (the earlier study of ileal SM [52]) is that in the pulmonary artery the RSK inhibitors are very effective in inhibiting the rate and lag phase of force development at 10 µM microcystin, a concentration at which the other inhibitors had no effect on the ileum. Based on our evidence for phosphorylation and activation of ERK1/2 and RSK, we conclude that in the pulmonary artery, unlike in the ileum, the force developed at 10 µM microcystin at pCa 9.0 reflects the ERK/RSK-mediated phosphorylation of RLC_20_. The residual microcystin-induced force in the presence of the RSK inhibitor reflects the activity of other Ca^2+^ independent kinases such as ZIPK consistent with our finding that ZIPK is not eluted from a γ-phosphate linked ATP resin by BI-D1870. Constitutively active ILK is unavailable for testing.

**Figure 8:**
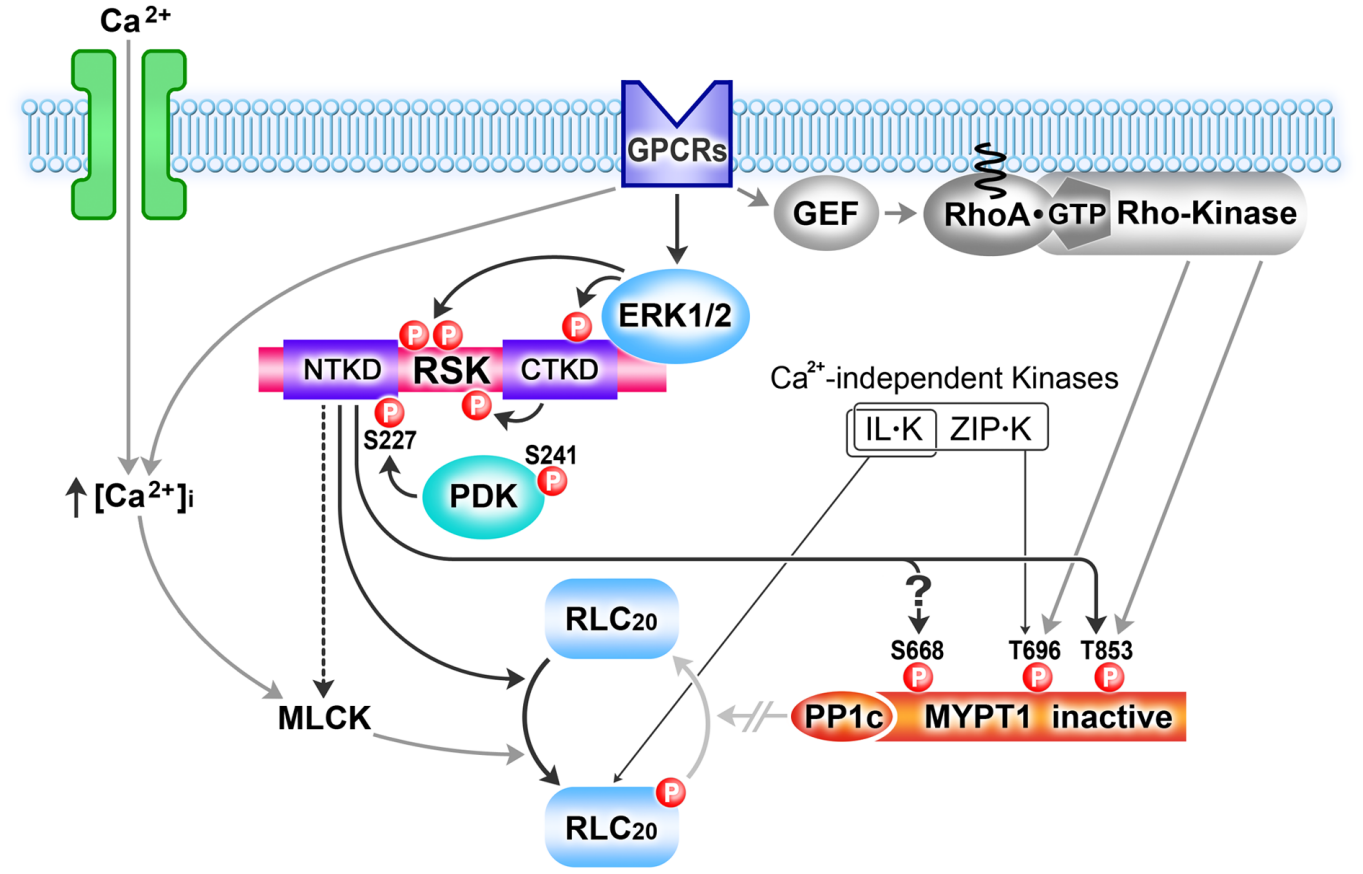
Ribosomal S6 kinase (RSK) signaling scheme for regulation of Ca^2+^-independendent contraction of smooth muscle. Agonists through GPCRs leads to activation of sequential ERK1/2 phosphorylations of RSK, subsequent RSK autophosphorylation by its C-terminal kinase domain (CTKD), the recruitment of phosphoinositide-dependent kinase 1 (PDK1) to this newly phosphorylated site, and finally PDK1-dependent phosphorylation at Ser227, with concomitant activation of the NTKD. Active RSK increases RLC_20_ phosphorylation, either directly or indirectly, as well as inhibitory phosphorylation of MYPT1 at Thr696/853 augmenting Rho/ROCK phosphorylation of these sites. Potential RSK phosphorylation of Ser668 on MYPT1 is also indicated.

Altogether, our findings demonstrate that the canonical upstream RSK activation signaling pathways established in other cell types are present and activated in SM, supporting a novel function for p90 ribosomal S6 kinases in the regulation of SM contractility (Figure 8). It remains to be shown whether the RSK signaling pathway also contributes to myosin-based motility of non-muscle cells and whether it contributes to deregulated contractility in diseases of SM.

## Supporting Information

Figure S1 (A) Western Blot analysis shows absence of cross-reactivity of anti-RSK1 and anti-RSK3 antibodies with RSK2. (B) Expression profile of RSK1 and RSK2 in rabbit and mouse smooth muscle tissues. Positive control: over-expressed truncated RSK2. (*: the quantity of uterus protein sample loaded for RSK2 is half of all other mouse samples. (C) Alignment of C-terminal part of human (h), mouse(m) and rabbit(rab) RSK isoforms used for the commercial antibody production. Note the perfect homology between species for given isoforms RSK1 and RSK2. Therefore, the presence of RSK1 protein in rabbit but not mouse smooth muscle tissues is not due to differences in antigen sequence.
(TIF)

Figure S2 Dose-response curves for RSK inhibitors (BI-D1870 and SL0101-1), PDK inhibitor (GSK2334470) and corresponding concentrations of the diluent, DMSO carried out in a-toxin permeabilized rabbit pulmonary artery SM partially contracted with pCa 6.7.
(TIF)
