# Supplementary figures and images for: The p90 Ribosomal S6 Kinase (RSK) Is a Mediator of Smooth Muscle Contractility
Source: PLoS One. 2013 Mar 13;8(3):e58703. doi: 10.1371/journal.pone.0058703 (PMC3596281; doi:10.1371/journal.pone.0058703)

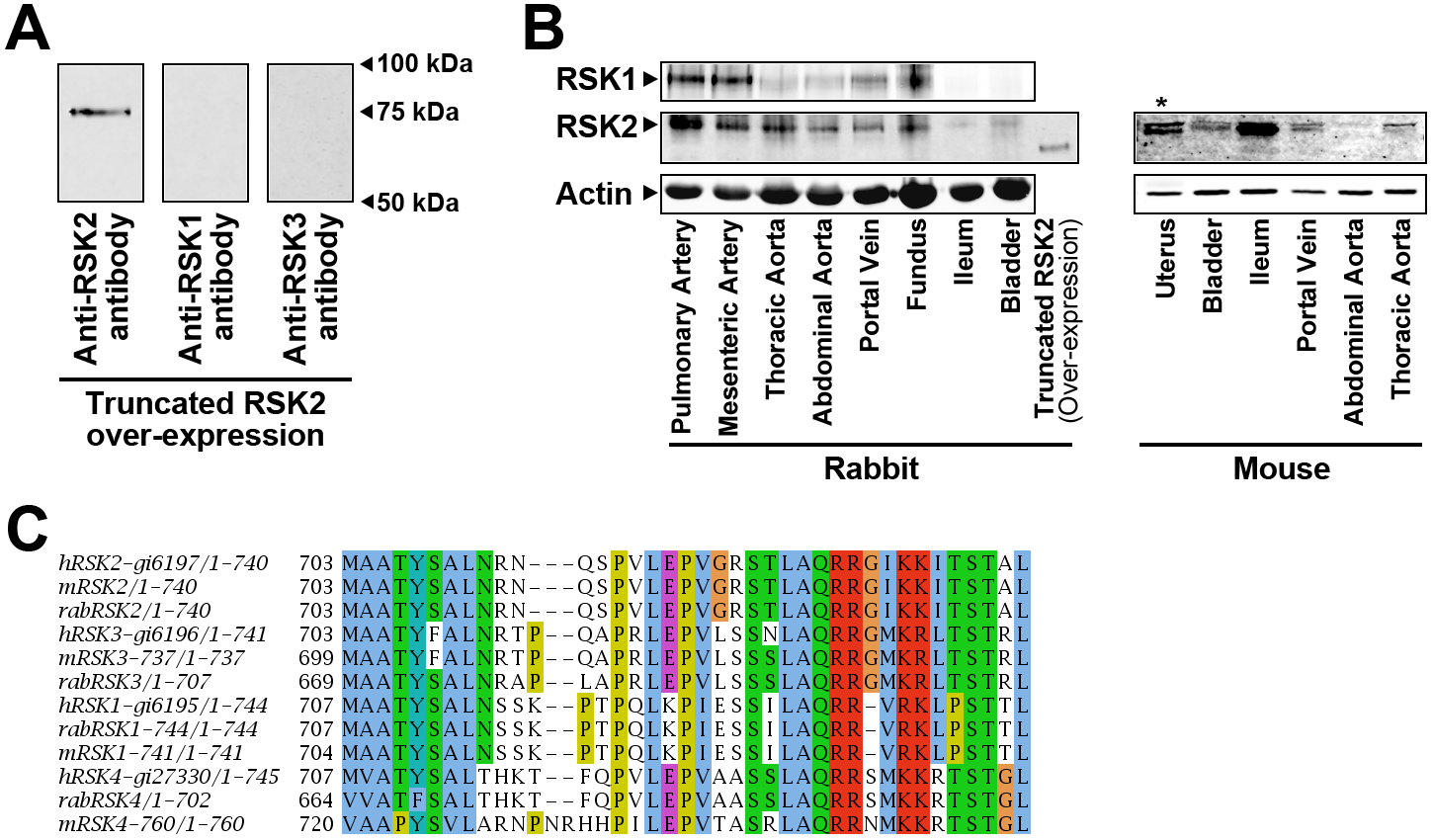

Supplement: Figure S1 — (A) Western Blot analysis shows absence of cross-reactivity of anti-RSK1 and anti-RSK3 antibodies with RSK2. (B) Expression profile of RSK1 and RSK2 in rabbit and mouse smooth muscle tissues. Positive control: over-expressed truncated RSK2. (*: the quantity of uterus protein sample loaded for RSK2 is half of all other mouse samples. (C) Alignment of C-terminal part of human (h), mouse(m) and rabbit(rab) RSK isoforms used for the commercial antibody production. Note the perfect homology between species for given isoforms RSK1 and RSK2. Therefore, the presence of RSK1 protein in rabbit but not mouse smooth muscle tissues is not due to differences in antigen sequence. (TIF) [file pone.0058703.s001.tif]

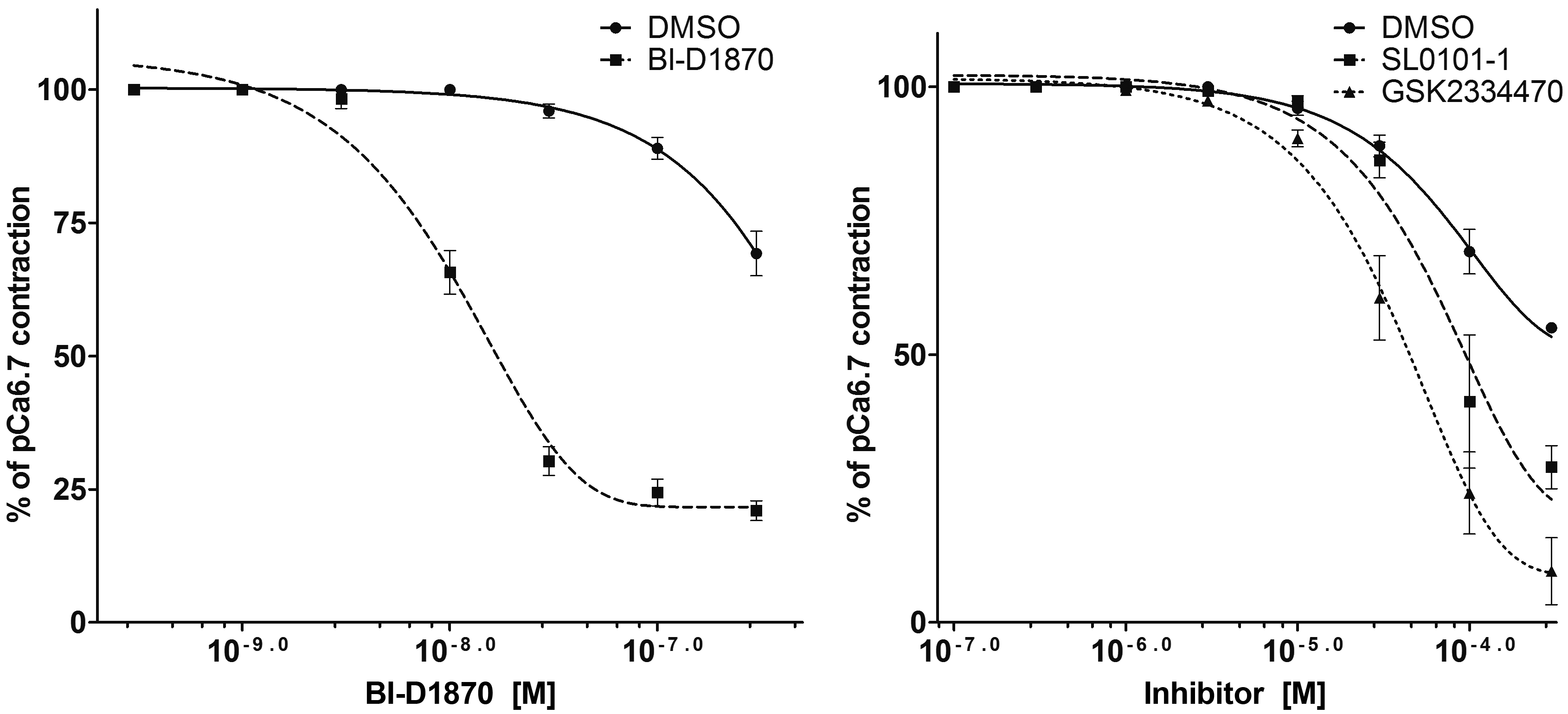

Supplement: Figure S2 — Dose-response curves for RSK inhibitors (BI-D1870 and SL0101-1), PDK inhibitor (GSK2334470) and corresponding concentrations of the diluent, DMSO carried out in a-toxin permeabilized rabbit pulmonary artery SM partially contracted with pCa 6.7. (TIF) [file pone.0058703.s002.tif]
